# Roles of SETD2 in Leukemia—Transcription, DNA-Damage, and Beyond

**DOI:** 10.3390/ijms20051029

**Published:** 2019-02-27

**Authors:** Anna Skucha, Jessica Ebner, Florian Grebien

**Affiliations:** 1 CeMM—Research Center for Molecular Medicine of the Austrian Academy of Sciences, Lazarettgasse 14, AKH BT 25.3, 1090 Vienna, Austria; an.skucha@gmail.com; 2 Ludwig Boltzmann Institute for Cancer Research, Waehringer Strasse 13A, 1090 Vienna, Austria; Jessica.Ebner@lbicr.lbg.ac.at; 3 Institute for Medical Biochemistry, University of Veterinary Medicine Vienna, Veterinaerplatz 1, 1210 Vienna, Austria

**Keywords:** SETD2, acute myeloid leukemia, mutations, histone modifications

## Abstract

The non-redundant histone methyltransferase SETD2 (SET domain containing 2; KMT3A) is responsible for tri-methylation of lysine 36 on histone H3 (H3K36me3). Presence of the H3K36me3 histone mark across the genome has been correlated with transcriptional activation and elongation, but also with the regulation of DNA mismatch repair, homologous recombination and alternative splicing. The role of SETD2 and the H3K36me3 histone mark in cancer is controversial. SETD2 is lost or mutated in various cancers, supporting a tumor suppressive role of the protein. Alterations in the SETD2 gene are also present in leukemia patients, where they are associated with aggressive disease and relapse. In line, heterozygous SETD2 loss caused chemotherapy resistance in leukemia cell lines and mouse models. In contrast, other studies indicate that SETD2 is critically required for the proliferation of leukemia cells. Thus, although studies of SETD2-dependent processes in cancer have contributed to a better understanding of the SETD2–H3K36me3 axis, many open questions remain regarding its specific role in leukemia. Here, we review the current literature about critical functions of SETD2 in the context of hematopoietic malignancies.

## 1. Introduction

Histone tails are subject to extensive posttranslational modification, such as methylation, acetylation, phosphorylation, ubiquitination and others, resulting in the establishment of the so-called “histone code”. The information encoded in the epigenetic modification of histones is critical for the regulation of a multitude of cellular processes, including transcription, DNA replication and DNA repair after damage. Thus, enzymes modulating these epigenetic processes play an important role in all stages of development and their dysregulation often leads to genetic disorders, including cancer. While the majority of histone modifications are associated with transcriptional control and related processes, the importance and functional consequences of many types of histone modifications remain incompletely understood. This is illustrated by the example of tri-methylation of histone H3 on lysine 36 (H3K36me3) by the methyltransferase SETD2. H3K36 tri-methylation by SETD2 has been linked to a plethora of critical pathways and processes, yet the functional implications of perturbed H3K36 tri-methylation by SETD2 mutations in cancer are still unclear. In this review, we will discuss the main functions of SETD2 and H3K36 tri-methylation and potential consequences of their dysregulation in hematopoietic malignancies.

SETD2 was first described in 1998 as a gene associated with Huntington disease [1]. Soon after, it was found to be expressed in human CD34+ hematopoietic stem and progenitor cells and to possess H3K36-specific histone methyltransferase activity [2]. Interestingly, mono- and di-methylation of H3K36 can be catalyzed by a number of promiscuous enzymes, including NSD1, NSD2 and NSD3 (Nuclear receptor-binding SET domain-containing proteins 1-3), ASH1L (Absent small and homeotic disks protein 1 homolog) and SMYD2 (SET and MYND domain containing 2), while SETD2 is the sole methyltransferase responsible for cellular H3K36 tri-methylation, using H3K36me2 as a substrate [3,4,5].

In general, all functional domains of SETD2 are highly conserved from yeast to humans [6,7]. Set2, the Saccharomyces cerevisiae homologue of the human SETD2 protein is responsible for all three steps of H3K36 methylation [8,9]. The catalytic SET (Suppressor of Variegation, Enhancer of zeste and Trithorax) domain is flanked an AWS (associated with SET) and a Post-SET domain. In addition, SETD2 contains a coiled–coiled (CC) and a WW (Tryptophan-Tryptophan) domain, which are mediating protein–protein interactions [6,7]. Via its functional SR1 (Set2 Rpb1 interacting) domain, SETD2 interacts with the hyper-phosphorylated C-terminal domain (CTD) of Rpb1, the largest subunit of RNA Pol II [10]. The N-terminus of human SETD2 comprises more than half of the protein sequence, but it is not evolutionarily conserved and it is not known if it harbors any functional domains [8,9].

Constitutive knockout of *Setd2* resulted in embryonic lethality at E10.5–E11.5 due to defects in the vascular architecture [11]. Setd2 deficiency in the hematopoietic system led to altered differentiation capacity of hematopoietic stem cells. *Setd2*-knockout mice showed reduced numbers of myeloid, lymphoid and megakaryocyte progenitor cells as well as a strong reduction in phenotypic and functional hematopoietic stem cells [12]. *Setd2*-deficient hematopoietic progenitors displayed elevated levels of replicative stress, which impairs their proliferative capacity. Consequently, *Setd2*-deficient hematopoietic progenitors exhibited increased genomic instability, accumulation of secondary mutations and malignant transformation [13]. In addition, hematopoietic stem cells isolated from *Setd2*-deficient mice displayed elevated rates of apoptosis, reduced multi-potent differentiation potential and consequently differentiated towards mature cell types [12].

## 2. *SETD2* Mutations in Hematological Malignancies

**Figure 1:**
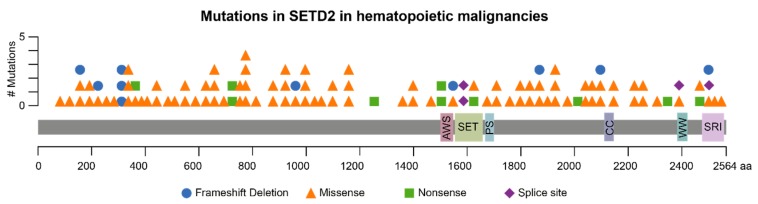
Schematic representation of *SETD2* mutations associated with hematopoietic malignancies. Mutations are represented according to type. The following hematopoietic malignancies are represented: Activated B-cell type, acute lymphoid leukemia, acute myeloid leukemia, B-lymphoblastic leukemia/lymphoma, chronic lymphocytic leukemia, diffuse large B-cell lymphoma and germinal center B-cell type leukemia. Duplicates were removed. SETD2 domains: AWS, associated with SET; SET, Su(var)3-9, enhancer-of-zeste trithorax; PS, post-SET; CC, coiled coil; WW, rsp5-domain; SRI, Set2 Rpb1 interacting. Mutation data were retrieved from cBioPortal (http://www.cbioportal.org) on 27 November 2018.

Mutations in the *SETD2* gene have been described in various human malignancies. Initially, *SETD2*-inactivating mutations have been found in 15% of patients with clear cell renal cell carcinoma (ccRCC) [14,15,16]. Alterations of *SETD2* were also identified in 30% of pediatric high-grade gliomas (HGGs) and colorectal cancer [17,18]. Mutations of *SETD2* were also found to be associated with hematopoietic malignances (Figure 1). In this cancer entity, mainly missense mutations can be found, which occur across the entire coding sequence. Focal deletions of *SETD2* were identified in 10% of patients suffering from early T-cell precursor acute lymphoblastic leukemia (ETP-ALL) [19]. Bi-allelic loss of *SETD2* was identified in mast cell leukemia (MCL) [20]. Moreover, *SETD2* mutations have been frequently identified in patients suffering from enteropathy-associated T-cell lymphoma and chronic lymphoblastic leukemia [21,22]. Finally, alterations in the *SETD2* gene were significantly enriched in relapsed pediatric acute lymphoblastic leukemia (ALL) patients, pointing towards a potential role of *SETD2* mutations in chemotherapy resistance [23]. This was recently confirmed, as heterozygous loss of SETD2 in leukemia resulted in resistance to DNA-damaging agents [24]. These findings and the high prevalence of *SETD2* mutations across different cancer entities strongly implied tumor suppressive functions of SETD2 and the corresponding H3K36me3 histone mark in cancer.

Several reports have characterized the role of normal and mutated *SETD2* in leukemia with MLL (Mixed Lineage Leukemia)-fusion genes. Zhu et al. described nonsense and frameshift mutations in *SETD2* in pediatric patients with MLL-rearrangements [25]. shRNA-mediated knockdown of SETD2 led to proliferative advantage, increased colony formation and accelerated leukemia development of fusion-protein expressing leukemia cells in vitro and in vivo, further establishing a tumor suppressive role of SETD2 in leukemia. Conversely, several genome-scale CRISPR (Clustered Regularly Interspaced Short Palindromic Repeats)/Cas9 screens identified *SETD2* as an essential gene in leukemia cells, proposing alternative functions of SETD2 in addition to its tumor suppressor role [26,27,28,29]. Using a domain-focused CRISPR/Cas9 mutagenesis approach, it was shown that the catalytic activity of SETD2 was essential, as mutagenesis of the *SETD2* SET domain impaired the proliferation of MLL-AF9-expressing leukemia cells [29]. In line with this, we recently found that shRNA- and CRISPR/Cas9-mediated loss of *SETD2* led to differentiation, enhanced DNA damage and apoptosis of acute myeloid leukemia (AML) cells harboring MLL-fusions in vitro and in vivo [30].

These observations indicate that heterozygous SETD2 loss, as frequently found in AML patients, accelerates leukemogenesis driven by the MLL-AF9 fusion protein, and perhaps also other oncogenic drivers. In contrast, complete SETD2 loss, as induced by homozygous deletion or near-complete loss-of-function-induced shRNAs or CRISPR/Cas9-mediated mutagenesis significantly delayed disease progression. These seemingly opposing observations imply that homo- versus heterozygous SETD2 loss has significantly different effects on leukemogenesis. As the majority of cancer patients present with heterozygous mutations in *SETD2*, it is conceivable that SETD2 functions as a haplo-insufficient tumor suppressor, while its homozygous loss impedes disease progression. Such a scenario might be of therapeutic relevance, as complete ablation of SETD2 may preferentially impact leukemia cells harboring heterozygous mutations of *SETD2*. However, homozygous depletion of *SETD2* in T-cells was associated with rapid expansion of the γδ-T-cell population [21]. This indicates that SETD2-dependent effects might be context-specific. Furthermore, it might be important to differentiate between effects that depend on the enzymatic activity of SETD2 (such as H3K36 methylation) and potential other molecular functions of SETD2 in the context of hematopoietic malignancies.

## 3. Mechanism of Action of SETD2 in Leukemia and Oncogenesis

SETD2 has been implicated in a number of cellular processes, many of which are dysregulated in cancer. The relative contribution of SETD2 to these molecular pathways is unclear, and we are only beginning to understand how dysbalanced SETD2 levels affect these processes in the context of a malignant cell. In the following, we will highlight the most important cellular functions of SETD2 and their potential links to leukemia.

### 3.1. SETD2-Dependent Regulation of the DNA Damage Response

**Figure 2:**
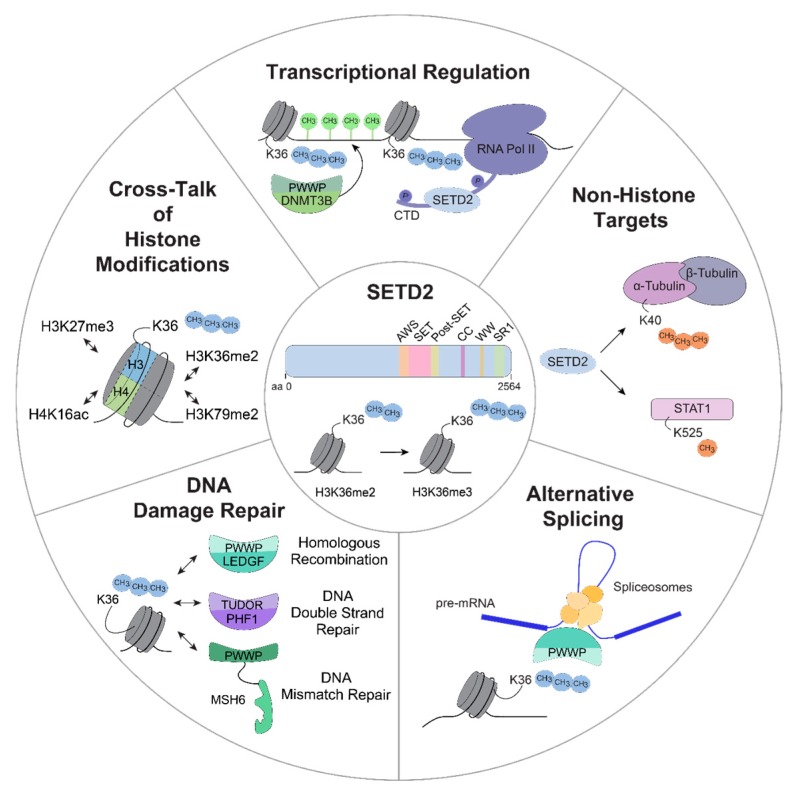
Schematic overview of potential functions of SETD2. SETD2-mediated H3K36 tri-methylation is involved in different cellular processes that are potentially associated with oncogenesis, including transcriptional regulation, methylation of non-histone targets, alternative splicing, DNA damage repair and an extensive crosstalk between H3K36me3 and other histone modifications. aa, amino acid; H3K36me2/me3, di-/tri-methylation of lysine 36 on histone H3; RNA Pol II, RNA polymerase II; CTD, carboxy terminal domain; DNMT3B, DNMT3B, DNA (cytosine-5-)-methyltransferase 3 beta; STAT1, signal transducer and activator of transcription 1; LEDGF, PC4 and SFRS1 interacting protein 1 (PSP1); PHF1, PHD finger protein 1; MSH6, MutSα homolog 6; H3K79me2, di-methylation of lysine 79 on histone H3; H3K27me3, tri-methylation of lysine 27 on histone H3; H4K16ac, acetylation of lysine 16 on histone H4.

The SETD2-dependent H3K36me3 mark is recognized by a number of epigenetic readers which bind this histone modification through chromodomains, such as PWWP (Proline-Tryptophan-Tryptophan-Proline) or Tudor (Figure 2) [3,31]. Loss of SETD2 was associated with altered DNA mismatch repair (MMR), as MSH6 (MutS homolog 6), a known component of the MutSα mismatch recognition complex, interacts with the H3K36me3 mark via its PWWP domain. Loss of SETD2-mediated H3K36 methylation as well as loss-of-function mutations of the MSH6 PWWP domain resulted in increased frequencies of spontaneous mutations, which are characteristic for MMR [32]. In line with this, LEDGF (Lens epithelium-derived growth factor, PSIP1), a known interaction partner of MLL and MLL-fusion proteins in acute leukemias utilizes its PWWP domain to bind to H3K36me3 and facilitates DNA-end resection during homologous recombination (HR). In addition, LEDGF was shown to recruit the DNA-damage repair machinery including RAD51, RPA (Replication protein A), and RBBP8 (CtBP-interacting protein, CtIP) to sites of damaged DNA [33,34]. The Tudor domain-containing protein PHF1 (PHD finger protein 1), a member of the Polycomb Repressor 2 (PRC2) complex, interacts with DNA double strand breaks marked with H3K36me3 and is involved in early events of the DNA damage response [31]. In this case, loss of SETD2-mediated H3K36 tri-methylation reduced PHF1 retention at double strand break sites, decreasing the efficiency of repair mechanisms. We found that loss of SETD2 resulted in increased DNA damage and activation of the tumor suppressor p53 and its target gene p21 [30]. Transcriptomic analysis identified enriched expression of DNA-damage-associated gene sets in *SETD2*-deficient cells. The same phenotype was observed in *SETD2*-deficient ccRCC cells. In this context, loss of SETD2 resulted in the accumulation of DNA damage and inefficient DNA damage repair. However, this was attributed to the inability of *SETD2*-deficient cells to efficiently activate p53 [35]. This controversy suggests that while observed phenotypes might be similar, the underlying molecular mechanisms might be tissue- and/or context specific. Knowing that SETD2 is part of a large multi-protein assembly nucleated by MLL-fusion proteins in the context of AML, it might be tempting to speculate that specific activities of SETD2 might be strongly dependent on the protein complex architecture in which it is embedded [30].

### 3.2. Cross-Talk between H3K36me3 and Other Histone Modifications

The importance of SETD2 in MLL-rearranged AML depends on chromatin targeting of the MLL complex by LEDGF, which interacts with methylated H3K36 via its PWWP domain [36]. Loss of H3K36me3 resulted in compromised chromatin binding of MLL-complexes and deregulation of MLL-fusion target gene expression. Down-regulation of SETD2 resulted in decreased binding of the MLL-AF9 fusion protein to promotors of known MLL target genes, such as *Hoxa9* (Homeobox protein Hox-A9) and *Meis1* (Homeobox protein Meis1, Myeloid ecotropic viral integration site 1), leading to reduced expression of these genes [30]. In another study, it was shown that loss of SETD2-dependent H3K36me3 induced elevated levels of the ASH1L-dependent H3K36me2 mark in MLL-AF9-expressing AML cells [37]. LEDGF was identified to preferentially bind to ASH1L-dependent H3K36me2-marked regions, thereby recruiting MLL-fusion-containing protein complexes to promoter regions. Thus, in this study, loss of SETD2-dependet H3K36me3 led to elevated levels of H3K36me2, enhancing the recruitment of LEDGF-containing MLL complexes to chromatin [37]. As the functional role of H3K36me3 remains elusive, further work is required to resolve these contradictory results.

Extensive crosstalk between the SETD2-dependent H3K36me3 mark and other histone marks has been reported (Figure 2). A strong correlation exists between the localization of the H3K36me3 mark and the DOT1L (Disruptor of telomere silencing protein 1-like)-dependent H3K79me2 mark in MLL-fusion expressing AML [30,38]. A dual H3K36me3-high-H3K79me2-high signature characterized MLL-target genes. shRNA-mediated knockdown of SETD2 resulted in a reduction of H3K36me3, but also in decreased H3K79me2 levels on MLL-target genes, indicating a strong interdependence of these histone marks. This is in contrast to data by Zhou et al. who found increased global H3K79me2 levels upon loss of *Setd2* [12]. This report identified a link between the loss of H3K36me3 and elevated levels of ASH1L, NSD1, NSD2 and NSD3 enzymes responsible for mono- and di-methylation of H3K36. Another study found that loss of SETD2 led to down-regulated expression of the tumor suppressor ASXL1 (Additional Sex Combs-Like protein 1) and an upregulation of ERG, resulting in accelerated leukemia development [39]. Even though the functional consequences of the enriched co-localization of the H3K36me3 and H3K79me2 histone modifications on MLL-target genes remain elusive and controversial, it indicates an important role of this dual signature in the biology of MLL-rearranged leukemia.

Besides the interplay of H3K36me3 with H3K79me2, it was reported that loss of H3K36me3 caused elevated levels of H3K27me3 in sarcomas [40]. This is in contrast to previous findings that suggest that binding of the Tudor domain-containing protein PHF1 to H3K36me3 might sterically preclude the ability of Polycomb complex PRC2 from deposition of the H3K27me3 mark [41]. An interplay between H3K36me3 and H4K16ac was also shown [42]. In this case, H3K36me3 triggers deposition of H4K16ac upon the induction of DNA damage.

Finally, aberrant regulation of epigenetic modulators can alter global gene expression patterns through loss of characteristic histone modifications patterns. Thus, distinct molecular lesions might converge on similar chromatin-based mechanisms to induce misregulation of overlapping gene sets to induce leukemia development. For instance, the gene expression profiles of *Setd2*-deficient hematopoietic cells were highly similar to global gene expression patterns of cells from *Tet2*/*Dnmt3a*-double knockout mice [13].

In summary, despite the presence of a large body of results suggesting the functional importance of epigenetic cross-talk in leukemia development, the mechanistic dependencies between H3K36me3 and other histone modifications remain unclear.

### 3.3. SETD2 in Alternative Splicing

In higher eukaryotes, post-translational modification of histone tails is associated with the regulation of alternative splicing. H3K36me3 was particularly implicated in this process, as intron-containing genes featured higher levels of the H3K36me3 mark than intron-less genes in mice and humans. In addition, genes containing constitutively included exons feature significantly higher H3K36me3 levels than genes with alternatively spliced ones (Figure 2) [43,44]. In agreement with this, shRNA-mediated loss of SETD2 led to reduced levels of exon inclusion in actively transcribed genes and a global switch of splice sites [45]. PWWP-domain containing proteins play a pivotal role in the regulation of this process. For instance, ZMYND11 (Zinc finger MYND-domain containing 11), BRPF1 (Bromodomain and PHD finger-containing 1) and MRG15 (MORF-related gene 15) bind to H3K36me3 and thereby either interact with components of the RNA splicing machinery or influence transcriptional regulation [45,46,47,48,49]. Likewise, the LEDGF-H3K36me3 interaction can modulate RNA splicing through LEDGF-dependent recruitment of spliceosome factors, such as SRSF1 (Serine/arginine-rich splicing factor 1) [50].

Given the strong evidence for a link between SETD2-dependent H3K36me3 and the regulation of alternative splicing, one may speculate that dysregulation of this process might contribute to malignant transformation. To date, however, the involvement of alternative splicing to leukemia development in the context of SETD2 has not been documented.

### 3.4. SETD2 in Transcriptional Regulation

The role of SETD2 in transcriptional elongation has been first described in yeast. The Set2 Rpb1 interacting (SRI) domain of the yeast Set2 protein interacts with the phosphorylated carboxy-terminal domain (CTD) of RNA polymerase II (RNA Pol II) [51]. A similar interaction was confirmed in mammalian cells [52,53]. RNA polymerase II is the integral part of the cellular machinery responsible for transcriptional regulation and interacts with a large number of accessory proteins, including structural proteins, epigenetic regulators, and transcription factors. The RNA Pol II CTD acts as a scaffold for the interaction with factors that regulate initiation, elongation and termination of transcription [54]. As SETD2 interacts with the CTD of elongating RNA Pol II, it was proposed that SETD2-dependent H3K36 tri-methylation across gene bodies prevents spurious intragenic initiation of transcription through the recruitment of a histone deacetylase complex [3]. In addition, SETD2 was demonstrated to play a role in nucleosome reorganization in bodies of transcribed genes after the passage of RNA Pol II machinery [55]. In this context, loss of SETD2 led to intragenic initiation of transcription through abnormal H3K36me3-dependent targeting of the FACT (Facilitates Chromatin Transcription) complex to chromatin, thus decreasing nucleosome occupancy on actively transcribed genes. As a result, down-regulation of SETD2 was associated with the induction of cryptic transcription of a large number of genes [55].

Interestingly, loss of SETD2 caused elevated levels of phosphorylated Serine 2 and Serine 5 in the RNA Pol II CTD, which led to higher levels of chromatin-association of elongating RNA Pol II with the *Myc* locus in the absence of *Setd2* [12]. Furthermore, a link between SETD2 and DNA methylation has been established, as DNMT3B (DNA (cytosine-5)-methyltransferase 3B) binds to the SETD2-dependent H3K36me3 mark via its PWWP-domain (Figure 2) [56]. It was suggested that DNMT3B-mediated methylation of gene bodies protects RNA Pol II from spurious re-initiation of transcription [57].

The global transcriptional effects of H3K36me3 loss might strongly depend on the tissue context. However, several studies found that SETD2 depletion only altered the expression of a small subset of genes rather than causing extensive changes in global gene expression patterns [30,57,58,59,60].

Taken together, chromatin binding of protein complexes involved in transcriptional regulation represents another cellular process reliant on SETD2-dependent deposition of H3K36me3. Deregulation of these processes caused by loss or mutation of *SETD2* might result in abnormal transcription that is associated with leukemia development.

### 3.5. Non-Histone Targets of SETD2

Interestingly, non-histone targets have been described to be subject to methylation by SETD2 as well (Figure 2). For instance, SETD2 binds and tri-methylates lysine 40 of α-Tubulin (α-TubK40me3) on mitotic microtubules [60]. In agreement, loss of SETD2 resulted in genomic instability and cytokinesis defects. This function of SETD2 might have a chromatin-independent impact on DNA damage processes and could contribute to the development of malignancies. Furthermore, SETD2 was found to mono-methylate lysine 525 (K525) of the transcription factor STAT1 (Signal transducer and activator of transcription 1) [61]. This modification was shown to enhance Interferon α signaling in hepatocytes, thus reinforcing antiviral immunity.

## 4. Therapeutic Opportunities

The concept of synthetic lethal interactions is of particular interest in cancer therapy, as certain genetic aberrations in cancer cells might expose specific vulnerabilities and resistance mechanisms against cancer drugs. Loss of SETD2 was shown to sensitize ccRCC cells to the PI3K (Phospoinositide 3-kinase) inhibitors TGX221 and AZD6482, thus decreasing the invasive potential of the targeted population [62]. Another study demonstrated that H3K36me3-deficient cancer cells are more sensitive to AZD1775-mediated inhibition of WEE1 kinase [58]. In MLL-fusion expressing leukemia, a combinatorial effect of SETD2 downregulation and pharmacological inhibition of DOT1L was observed [30]. DOT1L is a H3K79me2-specific methyltransferase and a known interactor of MLL-fusion proteins. Small molecule-mediated DOT1L inhibition proved to be a promising strategy in leukemia with MLL-fusion proteins. Targeting DOT1L using the small molecule inhibitor EPZ5676 (Pinometostat) in SETD2-mutated cells potentiated the effects of SETD2-downregulation on growth inhibition, induction of myeloid differentiation and apoptosis. Interestingly, loss of SETD2 and DOT1L inhibition also synergized in the induction of DNA damage [30]. These findings emphasize the need for the development of efficient SETD2 inhibitors. In an effort to characterize inhibitors of the enzymatic activity of SETD2, a drug screening approach revealed N-alkyl Sinefungin derivatives as most potent and selective SETD2 inhibitors when compared to a panel of methyltransferase enzymes [63]. However, given that Sinefungin is not cell permeable, this substance cannot be utilized for functional cellular studies. This raises an urge to identify novel, more potent agents or to find an alternative approach to target SETD2-dependent functions in cancer. The availability of specific small-molecule inhibitors of SETD2 function together with combinatorial approaches might represent a therapeutic strategy for patients suffering from leukemia and other SETD2-dependent malignancies.

## 5. Conclusions and Perspectives

Even though conflicting results have been obtained with regard to the molecular mechanisms involved, it has been unanimously recognized that SETD2 plays a pivotal role in hematopoietic malignancies. Given the diametrically opposed effects of heterozygous versus homozygous loss of SETD2 on leukemia development, it would be beneficial to gain a more profound knowledge about the mutational landscape of *SETD2* in hematopoietic cancers. In addition, a global view of the cellular SETD2 protein interaction network is still missing. The identification of SETD2 interaction partners will significantly enhance our knowledge about SETD2 and contribute to the clarification of the role of SETD2 in cancer. Overall, even though an unambiguous mechanism of action of SETD2 in cancer has not been revealed, recent reports have established the role of SETD2 in DNA damage repair, alternative splicing, regulation of transcription as well as a number of epigenetic dependencies. All these processes, when dysregulated, might contribute to the development of malignant diseases.
